# Paternal Psychological Stress After Detection of Fetal Anomaly During Pregnancy. A Prospective Longitudinal Observational Study

**DOI:** 10.3389/fpsyg.2020.01848

**Published:** 2020-07-29

**Authors:** Mona Bekkhus, Aurora Oftedal, Elizabeth Braithwaite, Guttorm Haugen, Anne Kaasen

**Affiliations:** ^1^Department of Psychology, PROMENTA Research Center, University of Oslo, Oslo, Norway; ^2^Faculty of Health Sciences, Oslo Metropolitan University, Oslo, Norway; ^3^Department of Psychology, Manchester Metropolitan University, Manchester, United Kingdom; ^4^Department of Fetal Medicine Oslo, University Hospital and Institute of Clinical Medicine University of Oslo, Oslo, Norway

**Keywords:** paternal stress, parental stress, fetal anomaly, pregnancy, prenatal ultrasound

## Abstract

**Background and Aims:**

Knowledge of carrying a fetus with a prenatal diagnosed anomaly may cause acute psychological stress to the parents. Most studies focus on maternal stress, yet fathers are often present at the ultrasound examinations and birth, and therefore may be affected, similarly, to the expectant mother. However, to date no existing studies have examined how detection of a fetal anomaly emotionally affects the expectant fathers throughout the pregnancy. Our aim was to longitudinally examine general health perceptions, social dysfunction and psychological distress in a subgroup of men where fetal anomaly was detected during pregnancy.

**Methods and Results:**

This study is part of the SOFUS study, a prospective, longitudinal, observational study. Participants were recruited when referred for an ultrasound examination conducted by a specialist in fetal medicine at Oslo University Hospital on suspicion of fetal malformation (study group). We examined differences between the men in the study group (*N* = 32) and a comparison group (*N* = 83) on the General Health Questionnaire (GHQ), Impact of Event Scale (IES) and Edinburgh Postnatal Depression Scale (EDPS) across four time points in pregnancy. Results from repeated measured ANOVA suggests that depression decreased over time among men in both groups (η^2^ = 0.15, *p* < 0.001). This effect was stronger in the study group, and differed from the comparison group (η^2^ = 0.08, *p* < 0.001). There was also a main effect of time on IES scores, which decreased over time for both men in the study group and in the comparison group (η^2^ = 0.32, *p* < 0.001). That is, men in the study group were higher on IES initially, but this effect decreased more in the study group than in the comparison group. Men in the study group and comparison group did not differ on perceived general health (GHQ: *p* = 0.864).

**Conclusion:**

Results suggests that detection of a fetal anomaly has implications for paternal mental health during pregnancy. Expectant fathers scored higher on EPDS and IES than the comparison group in the acute phase after detection of fetal anomaly, thus there is impetus to provide psychological support for fathers, as well as mothers, at this difficult time.

## Introduction

Fetal anomaly is a genetic or physical condition that affects the embryo or fetus, and can be defined as structural or functional anomalies. A fetal anomaly can vary from minor malformations to severe conditions that may lead to death or stillbirth (World Health Organisation [WHO], 2016). Ultrasound examination is considered an important part of maternity care. In Norway, all pregnant women are offered one free ultrasound examination (anomaly scan) at around gestational week 18. This examination is attended by 98% of the pregnant population (Reinar et al., 2008). In Norway, approximately 1200 babies per year are born after detection of a structural anomaly by ultrasound in pregnancy. The prognosis associated with these anomalies, however, may vary, and some mothers will have their pregnancy terminated following the detection of a fetal anomaly. For those who continue with their pregnancy, it has been emphasized that most couples consider it helpful to know the diagnosis in advance. However, knowledge of carrying a fetus with a prenatally diagnosed anomaly may also cause acute psychological stress to the parents.

In pregnancies without fetal anomaly, psychological distress for both mothers and fathers has been shown to increase over the course of pregnancy, and to be at the highest around the time of birth (Philpott et al., 2017). In a systematic review, Dikmen-Yildiz et al. (2017) found that after birth the prevalence of post-traumatic stress disorder (PTSD) in normal pregnancies was 4%. However, in contrast to uncomplicated pregnancies, detection of a fetal anomaly during pregnancy could increase psychological distress in early gestation. Indeed, the detection of a fetal anomaly in pregnancy has been associated with severe psychological distress in mothers (Korenromp et al., 2005; Kaasen et al., 2010). Elevated maternal psychological distress has been reported for severe and complex anomalies such as various congenital heart diseases, but also for abnormalities that are less severe and can be treated, such as cleft lip/palate. For example, in a recent study of 48 mothers carrying a fetus with complex congenital heart disease, maternal psychological distress was evident for 65% of the mothers, in contrast to 25% of those carrying a healthy fetus (Wu et al., 2020). Similarly, Rey-Bellet and Hohlfeld (2004) interviewed 29 expectant parents and found that most reacted with severe psychological shock in response to cleft lip/palate detection, but awareness that the deformity could be treated has been reported as a great relief (Nusbaum et al., 2008).

Daugirdaitè et al. (2015) examined findings from 48 studies after termination of pregnancy and found that post-traumatic stress (PTS) was greater than post-traumatic stress disorder (PTSD) after termination of pregnancy, but also that longer gestational age was associated with more PTSD. They also found that both PTS/PTSD decreased over time. However, detection of fetal anomaly that does not lead to termination of pregnancy may be associated with PTS/PTSD that persists throughout pregnancy. Detection of fetal anomaly is a psychological stressor (Côte-Arsenault and Denney-Koelsch, 2011) as for most couples the ultrasound examination is expected to provide information about a healthy baby. There is evidence that following such a stressor, stress levels may remain elevated throughout pregnancy (Nes et al., 2014) and that feelings of grief may persist until after birth (Hunfeld et al., 1999). Additionally, some expectant mothers develop severe PTSD (Davies et al., 2005). These findings are in contrast to research on major life stressors, that suggests that the impact of life stress usually has a short duration (Suh et al., 1996). Drawing on this research, one might expect a decrease in psychological reactions after detection of fetal anomaly. However, the predicted future severity of an anomaly detected *in utero* may vary throughout pregnancy as new diagnostic information could possibly be added to previous information. In previous research, we have, however, found that psychological distress in expectant mothers decreased from the time the fetal anomaly was diagnosed, to gestational week 36 (Kaasen et al., 2017).

To date, most studies have focused on maternal stress, yet fathers are often present at the ultrasound examinations, throughout pregnancy and birth of the child (Redshaw and Henderson, 2013). Additionally, fathers are increasingly involved in childrearing (Condon et al., 2004; Opondo et al., 2016). However, only during the last few decades have fathers been included in family research, and very little is known about how the detection of a fetal anomaly may impact the fathers’ psychological state (Statham et al., 2000). Therefore, they may also be at risk of psychological distress following the detection of a fetal anomaly (Ekelin et al., 2004) which may impact on their relationship with their partner (Halford et al., 2010) with additional implications for maternal mental health.

To our knowledge, just three studies have examined the impact of fetal anomaly detection in pregnancy on fathers’ psychological well-being. In a study by Skari et al. (2006) fathers reported lower symptoms of depression and post-traumatic stress, compared to the expectant mother. Another study examined psychological distress in couples who had terminated the pregnancy due to fetal anomaly, and reported a moderate difference between men and women, with women reporting higher symptoms of grief and post-traumatic stress, anxiety and depression (Korenromp et al., 2005). However, a key limitation of this study was that they examined grief and stress 2–7 years after the couple had terminated the pregnancy, and therefore findings are subject to recall bias. Another study by Kaasen et al. (2013) examined psychological distress in 155 men and women shortly after sonographic examinations. They compared couples where a fetal anomaly was detected to those with normal ultrasound findings. They found that men and women differed on their psychological response. However, they only included measures at one time point. Thus, a key limitation of the existing research is that it has not longitudinally examined psychological distress in expectant fathers over the course of pregnancy. Thus, in the current study, we use data from all four assessments, which was not available in previous publications from the SOFUS study. It is important to know whether the reported increases in psychological stress continue throughout pregnancy, and remains stable, but elevated. This is especially relevant as prenatal and postnatal depression are highly correlated in men (Skjothaug et al., 2015).

In summary, very little published research has examined psychological distress in expectant fathers after the detection of a fetal anomaly, and the existing research is limited to cross-sectional designs and, in one case, retrospective reports of psychological stress. This study aims to address gaps in the existing literature, with two key aims: (i) examine whether men with detection of a fetal anomaly score higher for psychological distress compared with a control group, (ii) test whether rates of psychological distress in these men may change throughout pregnancy.

## Materials and Methods

### Study Design and Participants

This study is part of the SOFUS study, a prospective, longitudinal, observational study.

The study was initiated by Oslo University Hospital, Rikshospitalet, and participants were recruited between May 2006 and February 2009. The SOFUS study is comprehensive and data was collected over 4 years (Kaasen et al., 2010, 2013). Data presented in the current study have not previously been published. The overall aim was to describe parental psychological and physiological reactions after ultrasound detected fetal anomaly.

**FIGURE 1:**
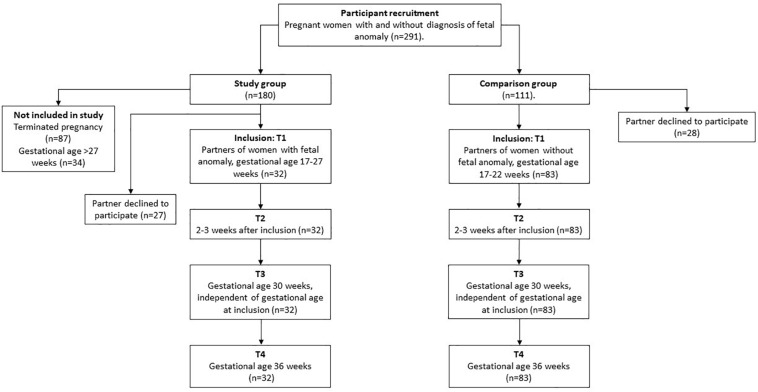
Flow chart of the study participants. Figure represents overview of the study group and comparison group across four time-points in pregnancy from inclusion.

The study sample consisted of expectant fathers and their pregnant partners receiving obstetric care at a tertiary perinatal care center (see Figure 1), flowchart for the study recruitment. Participants were recruited into one of two groups: the group with a fetal anomaly detected by ultrasound (study group) or the group with normal ultrasound findings (comparison group). The study group was recruited when referred for an ultrasound examination conducted by a specialist in fetal medicine at Oslo University Hospital, Rikshospitalet on suspicion of fetal malformation, at approximately week 18 of pregnancy. The comparison group was recruited at the routine ultrasound examination at approximately week 18 of pregnancy. During the recruitment period, 111 couples were eligible to participate, of these 28 men declined to participate in the study. Thus, the comparison group consisted of 83 expectant fathers, who participated in all study assessments. Of the 180 women (Kaasen et al., 2010) with a fetal anomaly, 27 fathers declined to participate and 87 pregnancies were terminated. In addition 34 men were included too late to participate on all four assessments, and therefore not included. There was no attrition during the four data collection points in pregnancy. It is important to note that these families were in regular contact with the hospital with close follow-up from staff. This may have contributed to their commitment to participate in the study. However, there were 1–3 missing datapoints (non-responses) on two subscales. The total sample consisted of 115 expectant fathers and their partners (*n* = 32 fathers in the study group and *n* = 83 fathers in the comparison group). The overall characteristics of the study sample are presented in Table 1, and suggests that fathers in the study and comparison group were different with respect to demographic variables (education, previous children, and gestational age at assessment).

**TABLE 1:** Characteristics for men and women in groups according to detected fetal anomaly or normal ultrasound scan.

	Fetal anomaly (*n* = 32)			No fetal anomaly (*n* = 83)			With and without fetal anomaly (*n* = 115)	
							Men vs. men	Women vs. women
	
	Men N (%)	Women N (%)	*P*-value	Men N (%)	Women N (%)	*P*-value	*P*-value	*P*-value
**Age**								
19–30 years	7 (22)	11 (34)	0.001	8 (10)	22 (27)	<0.001	0.216	0.437
31–35 years	11 (34)	14 (44)	0.001	34 (41)	33 (40)			
36–67 years	14 (44)	7 (22)	0.001	41 (49)	28 (34)			
**Education**								
≤Junior college	22 (31)	23 (37)	0.002	16 (19)	15 (18)	0.001	<0.001	0.002
>Junior college, <4 years	17 (53)	16 (50)	0.002	25 (31)	27 (33)	0.001	0.001	0.002
>Junior college, ≥4 years	7 (22)	5 (16)	0.002	29 (35)	40 (48)	0.001	0.001	0.002
Missing	8 (25)	11 (34)	0.002	38 (46)	1 (1)	0.001	0.001	0.002

**Previous children**								
Men and women no previous children	10 (31)			51 (61)			0.020	
Men previous children, women no previous children	1 (3)			3 (4)			0.020	
Men no previous children, women previous children	3 (9)			2 (2)			0.020	
Men and women previous children	18 (56)			27 (33)			0.020	
**Married or cohabitating**; Not cohabitating	30 (94); 2 (6)			83 (100); 0			0.133	
**Gestational age at assessment**								
18 weeks	7 (22)			14 (17)			0.003	
20 weeks	21 (66)			69 (83)			0.003	
22 weeks	4 (13)			0			0.003	
**Time from suspicion of fetal anomaly to examination at the referral center**								
2 days	26 (81)			n.a.			n.a.	
3–4 days	6 (19)			n.a.			n.a.	
5 days	0			n.a.			n.a.	
**Change in diagnosis/prognosis from T1 to T2**								
Improved	11 (34)			n.a.			n.a.	
Stable	20 (63)			n.a.			n.a.	
Worsening	1 (3)			n.a.			n.a.	
**Classification of severity**								
1; 2; 3; 4; 5	0 (0); 3 (9); 8 (25); 10 (31); 11 (34)			n.a.				

### Procedure

Assessments were performed in the acute phase following the diagnostic ultrasound examination (>24 h) and longitudinally with a total of four assessments during pregnancy. The study assessments consisted of psychometric distress measurements, in connection with consultation and ultrasound examination by a fetal medicine specialist. The first assessment (T1) was completed within 1–4 days following detection of a fetal anomaly or a normal finding on ultrasound examination. The second assessment (T2) was performed 2–3 weeks after T1. The third (T3) and fourth (T4) assessments were done at 30 and 36 weeks gestation, respectively. The median gestational age in the study group at recruitment was 18 weeks and 6 days (range 86–186 days). The variation in gestational age reflects that fetal anomaly may be detected at any time throughout pregnancy. In the comparison group the median gestational age was 19 weeks and 2 days (range 92–151 days).

#### Ultrasound Examination and Counseling

Fetal medicine specialists performed the ultrasound examinations. After the ultrasound examination, the fetal medicine specialist counseled all women in the study group, and specialists in neonatology, pediatric surgery, pediatric cardiology, neurosurgery, or medical genetics were additionally consulted, as needed. All fathers in this study were present at the ultrasound examination and counseling. The mothers with a diagnosed fetal anomaly received prenatal care with regular fetal assessments throughout the pregnancy, and the fathers were invited to attend all consultations.

#### Fetal Anomaly

The average sensitivity in the detection of a fetal anomaly, at the time of data sampling, was reported to be approximately 39% in a low-risk Norwegian population (Nakling and Backe, 2005). Fetal diagnoses at T1 were classified, according to Kaasen et al. (2010) with respect to severity and diagnostic or prognostic ambiguity at the time of recruitment. Three of the authors performed the classification, with strong inter-rater agreement (κ = 0.86) (Kaasen et al., 2010).

**A fetal anomaly was categorized as:**

(1) Lethal or serious with no available treatment, with or without prognostic ambiguity (e.g., acrania, skeletal dysplasia with small thorax, holoprosencephaly)
(2) Serious with available treatment, with prognostic ambiguity (e.g., myelomeningocele with hydrocephalus, hypoplastic left heart syndrome)
(3) Mild to moderate severity with available treatment, often with good result, but with prognostic ambiguity (e.g., bilateral clubfoot or cleft lip with no other markers, condition known to be associated with syndromes not apparent prenatally)
(4) Mild to moderate severity with available treatment, often with good result, without prognostic ambiguity (e.g., gastroschisis, unilateral clubfoot)
(5) Severity not classified; awaiting clarification. Prognosis highly dependent on the results of an invasive test (e.g., omphalocele, bilateral clubfoot with chromosomal soft markers), or a reliable diagnosis was not available at inclusion because of an incomplete ultrasound examination (e.g., maternal obesity)

“Not classified, anomaly awaiting clarification” corresponds to an inconclusive ultrasound examination at the referral center. Ten of the eleven pregnant women with this classification had an invasive fetal diagnostic test before T2, and they all received an answer before T2. One woman decided not to have an invasive test.

Throughout pregnancy, any changes in fetal diagnosis and/or prognosis were recorded as “improved,” “stable,” or “worse.” “Improved” signified that the fetal anomaly was determined to have less influence on the child’s future health compared with a previous assessment (e.g., following receipt of a normal karyotype). The diagnosis or prognosis was considered “worse” following the finding of an abnormal karyotype or following additional observations showing worsening of the fetal condition on repeated ultrasound examinations.

For those in the study group without clarification, the diagnosis/prognosis changed to a better prognosis in four fetuses (see Table 1), it worsened in one and was stable in six. In fetuses in the group “serious with available treatment but uncertain results (2)” and the group “serious to minor severity, usually good result following treatment, but with ambiguity” (3), all three and eight fetuses, respectively, were stable in prognosis throughout pregnancy from T1 to T4. In the group of fetuses “serious to minor severity, usually good result following treatment” (4) there was one fetus with improved prognosis, the rest were stable in prognosis (category 1 and 5). All these changes in prognosis happened between T1 and T2, and most were due to karyotyping of the fetus.

#### Measurements

*General Health Questionnaire (GHQ*) (Goldberg and Hillier, 1979) is a 28-item scale consisting of four seven-item subscales measuring social dysfunction, health perception (somatic symptoms), anxiety and severe depressive symptoms, during the preceding 2 weeks. The items were summarized based on a four-point Likert scale from 0 to 3. GHQ “case” scores were then dichotomised (i.e., much less “0”/less “1” vs. same “2”/better “3”) for each of the 28 items. In particular, the GHQ items 24 (“life is not worth living”), 25 (“considering ending my life”), 27 (“wished I was dead”), and 28 (“thinking about ending my life”) were used to assess suicidal ideation (based on a Likert score of 2–3 on any item) (Goldberg and Hillier, 1979). Other research groups have previously used the GHQ for assessment of distress in pregnancy, and it has previously been used in the Norwegian population (Skreden et al., 2010; Prady et al., 2013).

*Impact of Event Scale (IES)* is a 22-items questionnaire measuring emotional and behavioral responses to stressful events during the previous week (i.e., intrusion, avoidance, and arousal) (Horowitz et al., 1979). The IES-22 version used in this study includes six additional items measuring arousal and one additional item measuring intrusion as published by Weiss and Marmer (1997). The IES have been translated and are widely used in Norwegian populations (Skreden et al., 2010; Garthus-Niegel et al., 2013). Intrusion is characterized by unbidden thoughts and images, troubled dreams, strong waves of feelings, and repetitive behavior (related to the experience of knowing about the fetal condition, in the case of the study group). Avoidance is characterized by ideational constriction related to the fetal condition, denial of the consequences of the anomaly, blunted sensations, behavioral inhibition, and awareness of emotional numbness. Arousal measures distress-associated, psycho-physiological activation and is characterized by anger and irritability, a heightened startle response, concentration difficulties, and hypervigilance. The IES was rated on a 5 point Likert scale and summarized (Eid et al., 2009). The total sum score ranged from 0 to 40 for intrusion (Cronbach’s Alpha 0.81) and avoidance (Cronbach’s Alpha 0.81), and 0–30 for arousal (Cronbach’s Alpha 0.74). Intrusion and avoidance scores <9 is considered to be within the normal ranges, while 9–19 is considered a sub-threshold response. ≥20 indicates intrusion and avoidance responses of definite clinical importance. Previous studies have used the IES-22 for assessing stress in pregnancy (Qu et al., 2012; Rychik et al., 2013).

*Edinburgh Postnatal Depression Scale* (EPDS) (Cox et al., 1987) measured Depression by 10 items. Five of the items measure dysphoric mood, two measure anxiety, and one each measures guilt, suicidal ideas, and incidence of “not coping” experienced during the previous week. The EPDS has been validated for use in pregnancy and for men as well as women (Murray and Carothers, 1990). EPDS scores were calculated by summarizing 10 scores, ranging from 0 to 3, giving a possible total score of 30. EPDS total score ≥10 was associated with mild depressive symptoms and a score of ≥13 was used to identify moderate or more severe symptoms of depression (Eberhard-Gran et al., 2001). The EPDS item 10 (“the thought of harming myself has occurred to me”), was used to assess suicidal ideation. The questionnaires were completed at the hospital, and the participants were instructed to complete the questionnaires without discussing the questions with others.

### Statistical Analyses

For descriptive statistics, within and between the two groups of fathers, we used parametric or non-parametric analyses, and report both means and medians, when appropriate. We transformed continuous variables into categorical variables. Cut-off scores were based on statistical inspection of the distribution and cut-off scores for clinically relevant groups (Goldberg and Hillier, 1979). Longitudinal trends for the study and comparison group were examined using a 3 × repeated measures ANOVA with IES, GHQ and EPDS as the outcomes. Repeated measures ANOVA was selected over mixed linear models, because we do not have missing across the four time-points, and because we were interested in the longitudinal differences between the two groups across four repeated measures. Time was the within subjects factor and Group was the between-subjects factor. We used a Bonferroni correction to adjust for multiple comparisons, we considered a significant *p*-value of *p* = 0.05/3 (3 main analyses) = 0.017. For main outcomes with significant differences we also examined subscales. Finally, we adjusted for gestational age at inclusion, and previous children in the study group of fathers. These covariates have previously been found to influence psychological stress in mothers. All analyses were computed using SPSS version 26.0.

### Ethical Considerations

Written, informed consent was obtained before participation. The Regional Ethics Committee of Southern Norway approved the study December 21st 2005 and May 10th 2016 (Reference number S-05281, 2016/779/REK Sør-Øst). The Institutional Review Board approved the study. In accordance with the study protocol, any participant with a case score of “1” on at least one of the four GHQ items addressing suicidal ideation was contacted for clinical evaluation if necessary, and offered psychiatric assistance.

## Results

### Descriptive Comparisons Across the Main Outcomes

#### General Health Questionnaire

Independent sample Mann-Whitney *U* test showed that men who experienced fetal anomaly prenatally did not differ from the comparison group on overall general health (GHQ Sum case score) or on health perception across all four assessments (Table 2). However, the men in the study group reported more anxiety at all four assessments. They also reported higher social dysfunction as compared to the comparison group, except at T4 (Table 2). *The Impact of Event Scale*: For Time 1 and Time 2 the men in the study group were higher on intrusion, avoidance and arousal, than the comparison group. However, this difference was non-significant for intrusion and arousal at time points 3 and 4. Across all four assessments, fathers in the study group were more avoidant than the comparison group. *Edinburgh Postnatal Depression Scale:* The fathers in the study group also experienced more symptoms of depression, measured by EPDS at Time 1, 2 and 4, but no difference was found at Time 3.

**TABLE 2:** Psychometric scores in men with and without a fetal anomaly, assessed at four points in pregnancy.

			Study group *N* = 32			Comparison group *N* = 83			*P*-value
			Median (min-max)	Mean (*SD*)	N	Median (min-max)	Mean (*SD*)	N	
Time 1	GHQ	Sum Likert	17.0 (4–44)	18.1 (7.0)	32	14.0 (4–41)	15.5 (6.4)	83	0.019
		Heath perception	3.0 (1–17)	4.5 (3.5)	32	3.0 (1–21)	4.6 (3.6)	83	0.781
		Anxiety	6.0 (1–12)	5.8 (2.5)	32	3.0 (0–13)	4.0 (2.4)	83	<0.001
		Social dysfunction	7.0 (5–11)	7.4 (1.3)	31	7.0 (0–17)	6.6 (2.3)	83	0.009
		Depression	0.0 (0–6)	0.7 (1.3)	32	0.0 (0–4)	0.3 (0.7)	83	0.096
		Sum case score	2.0 (0–15)	2.6 (3.5)	32	0.0 (0–15)	1.8 (2.9)	83	0.190
	IES	Intrusion	12.5 (2–32)	13.7 (8.2)	32	6.0 (0–25)	7.4 (6.5)	83	<0.001
		Avoidance	5.0 (0–20)	6.1 (5.3)	32	0.0 (0–18)	1.8 (3.0)	83	<0.001
		Arousal	3.5 (0–18)	5.5 (4.8)	32	2.0 (0–11)	2.3 (2.5)	83	<0.001
	EPDS	Sum	5.0 (0–12)	4.6 (3.6)	32	1.0 (0–9)	1.4 (1.9)	83	<0.001
Time 2	GHQ	Sum likert	15.0 (6–37)	17.6 (7.3)	32	13.0 (5–37)	14.0 (5.4)	83	0.007
		Heath perception	4.0 (1–15)	5.3 (3.6)	32	3.0 (1–19)	4.1 (3.1)	83	0.075
		Anxiety	4.0 (1–12)	4.7 (2.8)	32	3.0 (0–9)	3.1 (2.0)	83	0.004
		Social dysfunction	7.0 (4–13)	7.1 (1.3)	32	7.0 (1–15)	6.7 (1.8)	82	0.021
		Depression	0.0 (0–4)	0.4 (1.0)	32	0.0 (0–6)	0.1 (0.7)	83	0.024
		Sum case score	0.0 (0–14)	2.4 (4.0)	32	0.0 (0–12)	1.1 (2.4)	83	0.101
	IES	Intrusion	7.0 (0–25)	9.6 (7.3)	31	4.0 (0–24)	5.2 (5.5)	82	0.001
		Avoidance	2.0 (0–22)	3.9 (5.1)	31	0.0 (0–10)	1.1 (2.0)	82	<0.001
		Arousal	3.0 (0–12)	3.5 (3.5)	31	1.0 (0–7)	1.7 (1.9)	82	0.006
	EPDS	Sum	1.0 (0–13)	2.8 (3.5)	32	0.0 (0–11)	1.2 (2.1)	83	0.014
Time 3	GHQ	Sum Likert	14.0 (8–37)	16.3 (6.0)	31	12.0 (6–30)	14.0 (5.6)	83	0.031
		Heath perception	4.0 (0–14)	4.6 (3.2)	31	3.0 (0–14)	3.9 (2.7)	83	0.330
		Anxiety	4.0 (0–12)	4.3 (2.2)	31	3.0 (0–10)	3.3 (2.4)	83	0.017
		Social dysfunction	7.0 (4–12)	7.3 (1.3)	31	7.0 (2–17)	6.6 (1.6)	83	0.016
		Depression	0.0 (0–2)	0.2 (0.5)	31	0.0 (0–3)	0.1 (0.4)	83	0.234
		Sum case score	0.0 (0.15)	1.9 (3.4)	31	0.0 (0–11)	1.0 (2.1)	83	0.232
	IES	Intrusion	5.0 (0–24)	6.0 (5.6)	31	3.0 (0–25)	4.4 (5.0)	83	0.072
		Avoidance	1.0 (0–19)	2.7 (3.9)	31	0.0 (0–9)	0.6 (1.4)	83	<0.001
		Arousal	2.0 (0–9)	2.5 (2.6)	30	1.0 (0–8)	1.6 (2.0)	83	0.087
	EPDS	Sum	1.0 (0–12)	1.7 (2.7)	30	0.0 (0–10)	1.1 (2.0)	83	0.143
Time 4	GHQ	Sum Likert	15.5 (7–37)	16.5 (6.4)	32	12.0 (2–34)	13.4 (5.8)	83	0.020
		Heath perception	4.0 (1–14)	4.6 (3.3)	32	3.0 (0–13)	3.8 (2.8)	83	0.169
		Anxiety	4.0 (0–13)	4.4 (3.0)	32	2.0 (0–10)	3.0 (2.4)	83	0.010
		Social dysfunction	7.0 (4–12)	7.2 (1.6)	32	7.0 (1–13)	6.6 (1.9)	82	0.169
		Depression	0.0 (0–2)	0.2 (0.6)	32	0.0 (0–5)	0.3 (0.7)	83	0.109
		Sum case score	0.0 (0–14)	2.4 (3.7)	32	0.0 (0–11)	1.2 (2.5)	83	0.123
	IES	Intrusion	5.5 (0–23)	6.0 (5.3)	32	2.0 (0–21)	4.3 (4.8)	82	0.053
		Avoidance	2.0 (0–12)	3.0 (4.3)	32	0.0 (0–13)	0.7 (1.9)	82	<0.001
		Arousal	2.0 (0–15)	3.0 (3.7)	32	1.0 (0–19)	2.1 (2.9)	82	0.360
	EPDS	Sum	2.0 (0–10)	2.5 (2.8)	31	0.0 (0–6)	0.9 (1.5)	82	0.001

*IES, Impact of Event Scale; GHQ, General Health Questionnaire; EPDS, Edinburgh Postnatal Depression Scale; SD, standard deviation; Time 1, at recruitment; Time 2, 2–3 weeks after recruitment; Time 3, at 30 gestational weeks; Time 4, at 36 gestational weeks. Comparisons tests used Independent sample Mann-Whitney U-Test.*

#### Repeated Measures ANOVA

**FIGURE 2:**
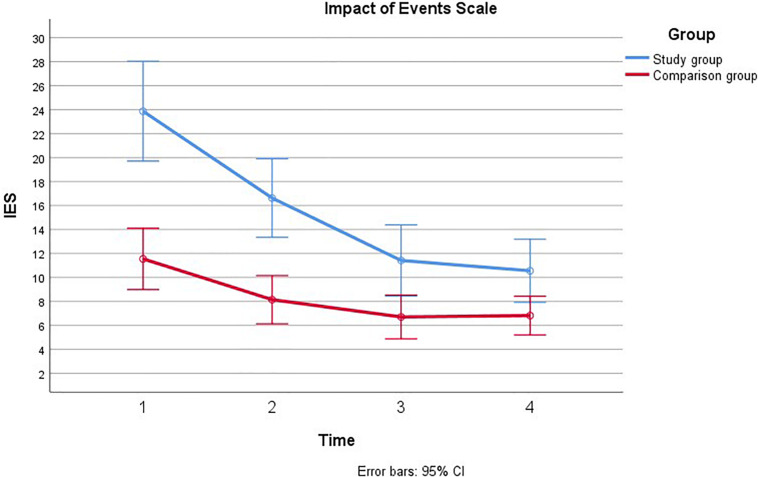
Comparison of General Health Questionnaire during pregnancy with and without a fetal anomaly. The figure presents General health in men in the study group (with fetal anomaly) and comparison group (normal ultrasound findings) at the four assessments, as measured by the General Health Questionnaire (GHQ).

**FIGURE 3:**
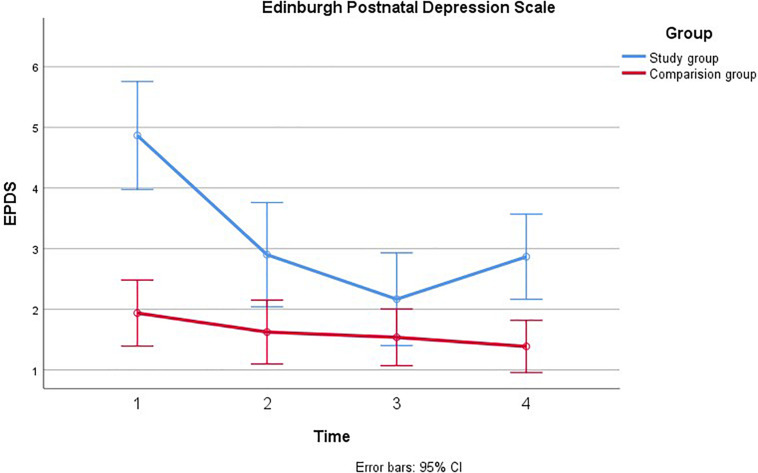
Comparison of paternal psychological distress during pregnancy with and without a fetal anomaly. The figure presents psychological distress in men in the study group (with fetal anomaly) and comparison group (normal ultrasound findings) at the four assessments, as measured by the Edinburgh Postnatal Depression Scale (EPDS).

**FIGURE 4:**
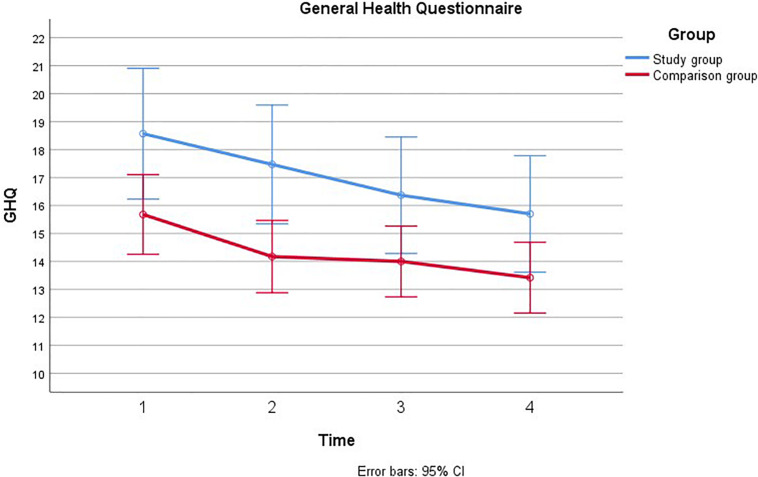
Comparison of paternal psychological distress during pregnancy with and without a fetal anomaly. The figure presents psychological distress in men in the study group (with fetal anomaly) and comparison group (normal ultrasound findings) at the four assessments, as measured by the Impact of Event Scale (IES).

First ANOVAs were examined for each of the covariates on each main outcome. There were no significant trends for education, paternal age, and time from suspicion of fetal anomaly. Therefore, we continued with the analyses adjusting for gestational age at inclusion and previous children that have been associated with stress in previous analyses (Kaasen et al., 2010). Separate repeated measures ANOVA’s were computed for each of the main outcomes across the four time-points in pregnancy (see Figures 1–4).

#### General Health Questionnaire

Mauchly’s test indicated that the assumption of sphericity had been violated, χ^2^(5) = 20.43, *p* = 0.001, therefore the degrees of freedom were corrected using Greenhouse-Geiser estimates of sphericity (*ε* = 0.88). There was a main effect of Time; GHQ decreased from T1 to T4 among men in both groups, [*F*(2.65, 118.90) = 4.57, *p* = 0.005, η^2^ = 0.04]. This decrease showed a linear trend [*F*(1, 301.54) = 9.65, *p* = 0.002, η^2^ = 0.08]. Men in the study group had higher GHQ scores than men in the comparison group [*F*(1, 160.43) = 8.74, *p* = 0.004, η^2^ = 0.07]. The main effect of group remained significant when adjusting for previous children and gestational age at inclusion. The main effect of time remained significant when adjusting for previous children, but not when adjusting for gestational age at inclusion, η^2^ = 0.011, *p* = 0.30. There was no significant time × group interaction for GHQ [*F*(2.65, 5.57) = 0.21, *p* = 0.864, η^2^ = 0.004].

#### The Impact of Event Scale

Mauchly’s test indicated that the assumption of sphericity had been violated, χ^2^(5) = 68.68, *p* < 0.001, therefore the degrees of freedom were corrected using Greenhouse-Geiser estimates of sphericity (*ε* = 0.69). There was a main effect of time, such that IES scores decreased over time for men both in the study group and in the comparison group [*F*(2.06, 2141.77) = 48.70, *p* < 0.001, η^2^ = 0.32]. Significant linear and quadratic trends were found for IES over time; [*F*(1, 3817.57) = 75.03, *p* < 0.001, η^2^ = 0.42] and [*F*(1, 520.12) = 21.55, *p* < 0.001, η^2^ = 0.17] for linear and quadratic, respectively. IES scores were also found to be higher among men in the study group than in the comparison group [*F*(1, 1125.72) = 19.34, *p* < 0.001, η^2^ = 0.16]. Furthermore, there was a significant group × time interaction, such that the study group had higher scores that decreased more rapidly over time than the scores in the comparison group [*F*(2.06, 470.45) = 10.70, *p* < 0.001, η^2^ = 0.09]. There was a significant linear trend for this interaction [*F*(1, 952.59) = 18.72, *p* < 0.001, η^2^ = 0.15]. These effects remained after adjusting for previous children, however, there was no longer a main effect of time on IES once gestational age at inclusion was adjusted, η^2^ = 0.06 *p* = 0.10. The time × group interaction was further broken down by comparing group means at each of the four time points using independent samples t-tests with a Bonferroni correction to adjust for multiple comparisons. There was a significant difference in IES between groups at T1, *t*_40.10_ = 4.11, *p* < 0.001, and at T2, *t*_37.93_ = 3.64, *p* = 0.004. Group differences were no longer significant at T3 and T4. We continued to examine each subscale, *intrusion, avoidance and arousal.* There was a significant group × time interaction effect for all subscales: intrusion [*F*(2.18, 146.90) = 6.95, *p* = 0.001, η^2^ = 0.06], avoidance [*F*(1.95, 25.03) = 4.33, *p* = 0.01, η^2^ = 0.04], and arousal [*F*(2.44, 21.11) = 4.85, *p* = 0.001, η^2^ = 0.05]. For all three subscales it appeared that IES scores were higher and decreased more rapidly in the study group relative to the comparison group.

#### Edinburgh Postnatal Depression Scale

Mauchly’s test indicated that the assumption of sphericity had been violated, χ^2^(5) = 14.84, *p* = 0.011, therefore the degrees of freedom were corrected using Greenhouse-Geiser estimates of sphericity (*ε* = 0.91). Depression was also found to decrease from T1 to T4 [*F*(2.73, 44.53) = 18.12, *p* < 0.001, η^2^ = 0.15]. Significant linear and quadratic trends were found for EPDS over time; [*F*(1, 88.07) = 33.62, *p* < 0.001, η^2^ = 0.23] and [*F*(1, 45.23) = 21.79, *p* < 0.001, η^2^ = 0.16] for linear and quadratic, respectively. There was also an effect of group, such that EPDS was higher among men in the study group than in the comparison group [*F*(1, 54.34) = 16.48, *p* < 0.001, η^2^ = 0.13]. In addition, there was a significant group × time interaction effect [*F*(2.74, 22.53) = 9.17, *p* < 0.001, η^2^ = 0.08]. This effect showed both linear [*F*(1, 32.91) = 12.56, *p* = 0.001, η^2^ = 0.10] and quadratic trends [*F*(1, 35.68) = 17.19, *p* < 0.001, η^2^ = 0.13]. In the study group, EPDS decreased from T1 to T3, with an uptake in depressive symptoms closer to time of birth (T4). In the comparison group, depression appeared relatively stable and lower than in the study group. These effects remained significant after adjusting for previous children, however, there was no main effect of time on depression once gestational age at inclusion was adjusted, η^2^ = 0.05 *p* = 0.08. To examine the group × time interaction effect we conducted four independent samples t-tests comparing the two groups at each time point, using a Bonferroni correction to adjust for multiple comparisons. Depression was significantly higher in the study group than the control group at T1, [*t*_38.19_ = 4.79, *p* < 0.001], but not at T2 or T3. At T4, depression increased again in the study group and became significantly higher than in the comparison group [*t*_38.21_ = 3.01, *p* < 0.02].

## Discussion

The aim of the current study was to prospectively examine, during pregnancy, psychological distress, measured by GHQ, IES, and EPDS in a subgroup of fathers and their partners after detection of fetal anomaly, and to compare their scores with a group without detection of fetal anomaly. We found that overall, men in the group with detected fetal anomaly were more anxious and experienced more psychological distress than men in the group with normal ultrasound findings. This psychological distress decreased throughout pregnancy from T1 to T4. Specifically, across all measures of distress, scores in the study group tended to be much higher than in the comparison group shortly after diagnosis, but then decreased throughout the remainder of the pregnancy to resemble those of men without detected fetal anomaly. One notable exception was that depression (EDPS) appeared to increase from T3 to T4 among men in the fetal anomaly group.

Although there was a mean difference on anxiety and social dysfunction measured by GHQ (see Table 2), we did not find that men in the study group and comparison group differed when compared longitudinally. Thus, it may be that the impact of detection of fetal anomaly diminishes more quickly for perceived general health than emotional distress. Perceived general health may also be a more broad and global measure. The fathers varied significantly in their initial levels of distress measured by the IES. This group difference remained after controlling for gestational age at inclusion; however, the effect of time did not. The results corroborate findings from previous cross-sectional studies indicating that there is an acute psychological response after detection of fetal anomaly, compared to healthy pregnancies. We also found a general pattern that this response declined through pregnancy. This is line with findings from a systematic review (Daugirdaitè et al., 2015) which found PTS and PTSD to decrease with time after termination of pregnancy. However, in our study this effect of time could be dependent on the severity of the diagnosis. For example, Dikmen-Yildiz et al. (2018) found longitudinal trajectories of PTSD after child birth to be predicted by further experience of trauma. Thus, if the stressor continues one could expect chronic levels of PTSD. Indeed, our findings suggest that men in the study group initially had higher levels of intrusion, arousal and avoidance as measured by the IES. However, at Time 4, the study group did not differ on intrusion and arousal. The IES measures event-specific distress, thus one may hypothesise that the fathers in the study group, with good follow-ups at the Hospital, had a better understanding of what awaits them. For example, Fonsesca et al. (2012) following parents of infants with life-threatening congenital anomalies from pregnancy to 6 months after birth, found that most parents of infants with life-threatening congenital anomalies recovered, but that 15% did not. However, recovery may also depend on severity of the diagnosis. In a large scale study, Nes et al. (2014) found that knowledge of carrying a fetus with Downs syndrome increased psychological stress in mothers. The present study did not allow for examining individual trajectories for similar subgroups. However, additional analyses found that classification of severity was positively associated with avoidance and depression. Thus, studies with larger populations could consider examining individual trajectories dependent on classification.

Similar to previous findings in expectant mothers, men in the study group experienced more distress than fathers with no fetal anomaly. Emotions may become elevated when a fetal anomaly has been detected during pregnancy, and thus result in increased symptoms of anxiety and depression. Studies from other research fields, suggest that the impact of stressful life events have a short duration (Suh et al., 1996) which support the decrease in symptoms after a 2–3 weeks period. We also found that depression (EPDS) may increase at T4, at 36 weeks of gestation close to the time of birth. This elevation in EPDS scores may reflect that stress related to fetal anomaly does not diminish as time goes by (as may be the case with some life events). Thus, one might think that for these fathers, the reality of expecting a baby with fetal anomalies and the potential complications this may have impact on their perceived stress. In fact, research suggests that stressors that cause changes in life circumstances have long-term effects on well-being and mental health (Luhmann et al., 2012). Our finding of increased symptoms of depression is also similar to research on expectant fathers experiencing pregnancies with normal ultrasound examinations. These studies have suggested that paternal depression tends to increase close to the time of birth and these symptoms have been found to persist into the postnatal period (Ramchandani et al., 2008; Skjothaug et al., 2018). This is important to consider as prenatal as well as postnatal depression in fathers has been found to have significant and long-lasting impact on child development (Ramchandani et al., 2008; Wilson and Durbin, 2010).

### Strengths and Limitations

The strength of this study includes a prospective longitudinal design, use of repeated assessments (four) based on the three standardized psychometric methods, and inclusion of a comparison group. We followed the participating fathers from the diagnostic ultrasound examination to 36 weeks gestation; thus we were able to follow changes in psychometric scores as well as in fetal diagnosis and prognosis. The paternal study group has to be considered as a selected group because their partner had either decided to continue with the pregnancy or the fetal anomaly did not give the legal option to terminate pregnancy.

Convenience sampling may have a potential for selection bias because included fathers are not a random sample of the total population. We aimed to minimize this problem by including men from a fixed date and only stopping inclusion when the workload made inclusion impossible. Another important note is the characteristics of the study group. The study group was characterized as those with pregnancies with fetal anomaly of such a character that most of them decided to continue their pregnancy and at the same time was diagnosed early allowing complete data collection (four times). Thus, although this design allows for an interesting perspective on dealing with acute and chronic stress, it is also a limitation as the study group have been selected based on these characteristics. For example, severity of the diagnosis may be important. However, although we adjusted for this in our analyses, this could not be examined when comparing the study and comparison group. It is also important to note that there was a wide variation of gestational ages in the study group at inclusion. In addition, other analyses, e.g., time-series, is not possible due to difference in gestational age at inclusion, the short interval between T1 and T2, and because gestational age has been found to influence stress levels (Kaasen et al., 2010). However, detection of fetal anomalies occurs throughout pregnancy, thus the present study reflects our patient population within fetal anomaly diagnostics.

Educational level differed between the groups and educational level may have an influence on psychological stress (Bjelland et al., 2008) and can possibly skew the results. Performing an adjusted ANOVA for educational level did not influence the stress level (GHQ, IES and EPDS). In addition we should note that the sample size is small. This not only limits the analyses available, but could also affect power to detect small effects. However, given the overall prevalence of this patient group, no other studies thus far have been able to follow expectant fathers longitudinally though pregnancy, where a fetal anomaly has been detected in pregnancy.

## Conclusion

In sum, expectant fathers in the study group scored higher on all measures than the comparison group in the acute phase after detection of fetal anomaly. Fathers had significantly higher symptoms of psychological stress, and PTSD symptoms, measured by IES following detection of anomaly than the comparison group. However, in both the study and comparison group, distress decreased from recruitment (gestational week approximately 19) to gestational week 30. At time-point four, the study group were somewhat higher on avoidance, but did not differ on intrusion or arousal (IES). However, EPDS depression increased toward gestational week 36. The implication of our findings suggests that there is a need to understand the long-term psychological impact for expectant fathers after detection of fetal anomaly. For some of these fathers, perceived psychological distress may elevate toward the time of birth.

To date, prenatal care, to a great extent, tends to focus on the mother. At first this may seem appropriate as findings often suggest that mothers tend to show more symptoms of psychological distress compared to their partner. However, our findings suggest that expectant fathers experiencing fetal anomaly also are at risk for psychological difficulties. Thus, paternal prenatal care is also important, to possibly prevent paternal depression in the prenatal and postnatal period (Skjothaug et al., 2018).

In sum, our findings suggest that for these fathers, expecting a baby with fetal anomaly is associated with psychological distress. In addition, for some of these fathers, this knowledge is associated with persistent stress that will impact on their life circumstances. Thus, these fathers need social support, and information about the pregnancy and forthcoming childbirth in a similar way as the expectant mothers.

Follow-up studies are needed to examine whether psychological distress, anxiety and depression decrease or increase in the postnatal period for expectant fathers with prenatally detected fetal anomaly.

## Data Availability Statement

The datasets generated for this study are available on request to the corresponding author.

## Ethics Statement

The studies involving human participants were reviewed and approved by the Regional Ethics Committee of Southern Norway approved the study December 21st 2005 and May 10th 2016 (Reference number S-05281, 2016/779/REK Sør-Øst). The patients/participants provided their written informed consent to participate in this study. Written informed consent was obtained from the individual(s) for the publication of any potentially identifiable images or data included in this article.

## Author Contributions

GH and AK planned and conducted the data collection for the SOFUS study. MB wrote the introduction and discussion and together with AK developed the research question and prepared the first draft of the manuscript. AO conducted additional analyses in the revised manuscript and worked on the datafiles. MB revised the manuscript. GH, AO, EB, and AK contributed to the interpretation of the data and analyses for the work. All authors revised the manuscript critically and approved the version to be published.

## Conflict of Interest

The authors declare that the research was conducted in the absence of any commercial or financial relationships that could be construed as a potential conflict of interest.
